# Differential weighting of lion vocalizations and oxpecker alarm calls in giraffes

**DOI:** 10.1093/beheco/arag099

**Published:** 2026-08-20

**Authors:** Anton Baotic

**Affiliations:** Acoustics Research Institute, Austrian Academy of Sciences, 1010 Vienna, Austria

**Keywords:** antipredator decision-making, heterospecific alarm cues, eavesdropping, predator signals, vigilance behavior, giraffe

## Abstract

Prey often assess predation risk using multiple sources of information that differ in immediacy and reliability. How prey differentially weight direct predator-associated cues and heterospecific alarm cues remains a central question in behavioral ecology. To investigate this, playback experiments were conducted on free-ranging, predator-experienced Southern giraffes (*Giraffa giraffa*), using lion (*Panthera leo*) vocalizations and red-billed oxpecker (*Buphagus erythrorhynchus*) alarm calls presented in different positions within sequential playback trials. Lion vocalizations consistently elicited strong vigilance responses regardless of playback presentation order, indicating that giraffes assigned greater behavioral weight to direct predator-associated information. Giraffes also responded to oxpecker alarm calls, although responses were generally weaker and more variable than responses to lion vocalizations, suggesting that indirect heterospecific cues were weighted less strongly than direct predator cues. Evidence that preceding playback exposure strongly influenced subsequent vigilance responses was limited, as lion vocalizations continued to elicit stronger responses than oxpecker alarm calls across successive playbacks. Females additionally exhibited generally stronger vigilance responses than males across playback conditions. Together, these findings suggest that giraffes differentially weight multiple sources of risk-related acoustic information during antipredator responses.

## Teaser

Not all warning sounds mean the same thing to giraffes. Lion vocalizations triggered stronger vigilance responses than alarm calls from red-billed oxpeckers, while previous playback exposure had only limited influence on later responses. Female giraffes also responded more strongly than males. These findings suggest that giraffes differentially weigh direct predator cues and indirect warning signals when assessing danger, highlighting how large mammals integrate multiple acoustic information sources to evaluate potential predation risk under natural conditions.

## Introduction

### Direct and indirect acoustic risk cues

Predation risk is a pervasive selective force shaping animal behavior, requiring prey to balance survival against competing demands such as feeding (Lima and Dill 1990; Brown and Chivers 2005; Caro 2005). Animals often rely on multiple sources of information to assess danger, including direct predator cues and heterospecific alarm calls (Dall et al. 2005; Goodale and Ruxton 2019; Magrath et al. 2020). These information sources differ in immediacy and reliability. Direct predator cues, such as predator vocalizations, provide immediate evidence of predator presence, whereas heterospecific alarm calls provide indirect information whose relevance may depend on ecological context, prior experience, and the reliability of the signaler (Magrath et al. 2015; Goodale et al. 2017; Palmer and Gross 2018; Magrath et al. 2020). Because antipredator responses are costly, prey are expected to adjust vigilance according to the perceived significance of available cues (McNamara and Houston 1986; Preisser et al. 2005; Stephens 2008).

Direct predator-associated acoustic cues often elicit strong and predictable antipredator responses across prey species. Lion vocalizations, for example, can rapidly increase vigilance and defensive behavior in African herbivores despite not necessarily indicating immediate hunting activity (Creel et al. 2014; Rigoudy et al. 2022). By contrast, responses to heterospecific alarm calls may be more variable because such signals can occur across a broader range of disturbance contexts and may provide less predator-specific information (Magrath et al. 2015; Turner et al. 2023). Many animals obtain information about danger by “eavesdropping” on alarm calls produced by other species, thereby exploiting publicly available information about predators or disturbances (Goodale et al. 2017; Magrath et al. 2020). Antipredator responses are often graded according to the perceived immediacy and reliability of available cues rather than reflecting fixed reaction patterns (Curé et al. 2015). Consequently, responses to heterospecific alarm calls may vary according to prior experience, ecological conditions, and concurrent information about risk (Lea et al. 2008; Makin et al. 2019). However, how prey prioritize indirect heterospecific alarm cues relative to direct predator vocalizations when both cue types are encountered sequentially within the same behavioral context remains comparatively understudied.

### Giraffe–oxpecker associations and alarm calls

Giraffes (*Giraffa* spp.) offer a biologically relevant system for examining how prey respond to direct and indirect risk cues. Although adult giraffes are capable of effective defense and are less vulnerable than juveniles, lion predation on giraffes is nevertheless well documented, particularly during vulnerable situations such as drinking, resting, or ambush encounters (Hayward and Kerley 2005; Valeix et al. 2009b; Périquet et al. 2010; Annear et al. 2023). Lions therefore remain an important source of predation risk influencing giraffe vigilance behavior. At the same time, giraffes maintain persistent ecological associations with red-billed oxpeckers (*Buphagus erythrorhynchus*), birds that forage on large mammalian hosts and frequently occupy positions that allow early detection of environmental disturbances (Weeks 2000; Nunn et al. 2011; Plantan et al. 2013). These associations may provide giraffes with benefits such as ectoparasite removal and early detection of potential threats, while also creating repeated exposure to oxpecker vocalizations.

Oxpeckers produce sharp hissing-like “ksss” calls during vigilance and disturbance contexts (Maclean et al. 1988). These calls are not predator-specific and may occur in response to a variety of disturbance or vigilance contexts, including approaching predators, large mammals, and human activity. Because oxpeckers frequently occupy elevated positions on giraffes and other large mammals, their alarm vocalizations may provide hosts with indirect early-warning information about elevated local risk. Consistent with this interpretation, oxpecker alarm calls have long been suggested to provide host species with early information about potential threats (Van Someren 1951; Leuthold 1977), and experimental work has demonstrated their alarm function in other systems, including rhinos (Plotz and Linklater 2020).

Recent playback studies further show that giraffes respond robustly to oxpecker alarm calls even in the absence of oxpeckers, indicating that these calls function as biologically meaningful risk cues (Baotic and Szipl 2025b). Giraffes also exhibit stronger vigilance responses to lion vocalizations in predator-experienced populations than in predator-free populations (Baotic and Szipl 2025a). Together, these findings suggest that giraffes use both predator-derived and heterospecific information when assessing potential danger. However, previous studies examined these cue types separately, leaving unresolved how giraffes respond when direct predator vocalizations and heterospecific alarm cues are encountered sequentially within the same behavioral context.

### Study aims and predictions

The present study addressed this question by presenting lion vocalizations and red-billed oxpecker alarm calls in different sequential orders to free-ranging giraffes. By varying cue order, the study tested whether giraffe vigilance responses primarily reflected prioritization of direct predator-associated information or whether responses to acoustic risk cues were influenced by preceding acoustic context.

Two alternative predictions were formulated. First, if giraffes prioritize direct predator cues because they provide more immediate and reliable information about danger, lion vocalizations should consistently elicit stronger vigilance responses than oxpecker alarm calls regardless of playback position. Second, if responses to heterospecific alarm calls are context sensitive, oxpecker alarm calls presented after lion vocalizations should elicit stronger vigilance responses than oxpecker alarms presented first. Because lion vocalizations represent direct indicators of predator presence, responses to lion calls were expected to remain comparatively stable across sequence positions. Finally, it was predicted that contextual effects would primarily influence vigilance response magnitude rather than the speed of initial response initiation. By directly comparing responses to heterospecific alarm cues and predator vocalizations within a single experimental framework, this study examines how a large herbivore evaluates multiple sources of risk-related information under natural conditions.

## Materials and methods

### Study site and subjects

Fieldwork was carried out from July to September 2024 at Lapalala Wilderness, a 48,000-ha private game reserve in Limpopo Province, South Africa. The study area comprises a mosaic of savanna and woodland habitats and supports a population of approximately 190 free-ranging Southern giraffes (*Giraffa giraffa*), based on a 2022 census. A pride of 15 lions (*Panthera leo*) was present at the time of the study. Lions were reintroduced into the reserve between 2019 and 2020 and have since established residency with confirmed records of lion predation on giraffes (Annear et al. 2023). The experimental sample included 35 adult giraffes, consisting of 19 males and 16 females. Sex and age class were assessed using external morphological traits according to the criteria of Muller et al. (2022). Identification of individual giraffes was based on their unique coat patterns, a widely accepted and reliable feature for distinguishing individuals in the wild (Bolger et al. 2012; Gholami et al. 2026).

### Playback stimuli

Acoustic playback stimuli were selected to represent 3 biologically distinct categories: lion roar-grunt vocalizations as direct predator cues, red-billed oxpecker alarm calls as heterospecific warning signals, and ring-necked dove (*Streptopelia capicola*) coo calls as a nonalarm control. Stimulus selection and preparation followed protocols established in previous giraffe playback studies (Baotic and Szipl 2025a, 2025b), although all exemplars and playback sequences used here were newly assembled for the present experiment.

Bird calls were recorded in the field in South Africa and supplemented with publicly available recordings from Xeno-canto and collaborators (see Acknowledgements). Lion vocalizations were sourced from archival recordings and collaborators (see Acknowledgements) to maximize stimulus diversity. All recordings were visually inspected using spectrograms in Praat (Boersma and Weenink 2023) to exclude overlapping calls, clipping, excessive background noise, and other recording artifacts. Retained recordings were subsequently processed using the workflow established in previous giraffe playback studies (Baotic and Szipl 2025a, 2025b). Signal processing included background-noise reduction in Audacity (Audacity Team 2023) followed by species-specific spectral filtering and amplitude normalization in Praat while preserving biologically relevant acoustic structure (Baker and Logue 2007; Benedict et al. 2021). Detailed recording procedures, equipment specifications, recording sources, software versions, and processing parameters are provided in the Methods S1.

Because species-specific playback stimuli differed markedly in their natural amplitudes, playback level was standardized across treatments to ensure comparable acoustic presentation across experimental conditions. The objective of the playback design was to compare behavioral responses to cue identity rather than to recreate absolute natural source amplitudes, which differ substantially between species. Standardizing playback amplitude (ie, sound pressure level, SPL) therefore minimized confounding effects of broadcast level while allowing ecologically interpretable comparisons of vigilance responses across stimulus categories. Playback amplitude was calibrated to 85 dB SPL (Z-weighted) at 1 m using an NTI AL1 SPL meter and MiniSPL microphone. The selected playback level ensured sufficient signal detectability while remaining within the range reported for comparable playback studies and available SPL measurements. Further justification of playback level selection, including comparisons with published SPL measurements, is provided in Methods S1.

Each stimulus sequence was constructed to reflect species-typical vocal behavior and bout structure. Lion sequences consisted of repeated roar-grunt combinations, oxpecker sequences reflected the short “ksss” alarm-call structure typically produced during vigilance or disturbance contexts, and dove sequences consisted of naturally spaced coo calls. Prior to field experiments, visual inspection of propagation trials at distances ranging from 25 to 150 m confirmed that the principal temporal and spectral characteristics of all playback categories remained visually distinguishable across the range of playback distances used during experiments. Spectrograms of these propagation trials are provided in the Figure S1 for lion calls, Figure S2 for oxpeckers and Figure S3 for doves.

### Playback package design

Prior to fieldwork, 28 playback packages were assembled, each comprising one lion, one oxpecker, and one dove sequence presented in a predetermined order. Within each playback sequence, individual stimulus exemplars were randomly selected from larger stimulus pools to preserve natural vocal variation while maintaining species-typical sequence structure. Playback order was designed to be balanced prior to fieldwork to ensure even representation of sequence conditions across tested individuals, and each playback sequence was assigned a unique stimulus identity code to allow stimulus identity to be incorporated into subsequent statistical analyses. Because field conditions occasionally prevented completion of playback packages, complete balance among first, second, and third playback positions was not fully retained in the final dataset (Table S2). Playback order was therefore included in all relevant statistical models to account for potential sequence-related variation. A total of 35 giraffes were tested, requiring some playback packages to be reused. However, no package was presented to more than 2 focal individuals. Detailed information on stimulus pools, sequence composition, and playback package construction is provided in Methods S2.

### Experimental procedure

Playback trials were conducted between 08:30 and 17:00 under stable weather and lighting conditions to ensure consistency across trials and to avoid periods of peak predator activity. Giraffes were located in the field using a DJI Mini 4 Pro drone flown at 40 to 70 m altitude, after which the observer approached the focal animal by vehicle. Playback trials focused on focal individuals that, based on the observer's visual assessment under natural browsing conditions, were not engaged in immediate conspecific interactions. In mixed groups, trials were typically conducted when herd members had dispersed while feeding, often resulting in interrupted visual contact between individuals due to vegetation structure. Trials were initiated only when a focal individual was stationary and engaged in relaxed feeding behavior. Because nearby browsing giraffes could occasionally remain obscured by dense vegetation, an observer-based visibility buffer of approximately 25 m around the focal individual was used as a practical field criterion to reduce the likelihood of immediate unseen neighbor-driven social influences on vigilance responses. Because giraffe vigilance behavior may be influenced by local social environment, nearest-neighbor distances and group composition would ideally have been incorporated into analyses. However, these variables could not be systematically quantified under dense vegetation conditions and were therefore not included as covariates. No other vehicles were present in the area during trials.

Prior to playback, the loudspeaker (JBL-EON Compact; 37.5 Hz–20 kHz) was mounted on a tripod approximately 1.5 m above ground level, concealed behind vegetation and oriented toward the focal individual at a distance of 33 to 135 m. Playback stimuli were broadcast using an Apple iPhone 14 Pro. The loudspeaker position remained fixed throughout each playback package so that successive playback stimuli were generally presented from the same distance to the focal giraffe, aside from minor movements of the animal during the trial. Playback distance was measured using a Nikon Prostaff 1000 laser rangefinder and recorded for each trial. The observer remained inside the stationary vehicle for the entire duration of each trial to minimize human disturbance and for personal safety due to the presence of free-ranging lions in the study area. Playback was initiated only after the focal giraffe had remained stationary and engaged in uninterrupted relaxed feeding behavior for at least 10 s without obvious vigilance escalation, locomotion, or external disturbance. Each playback package was delivered only once per individual and represented a single decision context, allowing assessment of short-term cue weighting independent of learning across trials. All trials were video recorded in high definition for later behavioral analysis.

Behavioral observations for each playback followed a 3-phase structure: baseline (prestimulus), response (during stimulus presentation), and post-response (resumption of feeding after playback). If the focal animal moved out of view, interrupted relaxed feeding behavior, or relocated between playback sequences, that is, after completion of a stimulus presentation but before the next sequence could be started, the trial was temporarily paused and resumed if the giraffe returned to calm foraging within the observation period. No giraffe ever moved out of sight during the response phase of a stimulus sequence. If visual contact was permanently lost before all sequences in the package could be delivered, the trial was considered incomplete. In 3 cases, the final (dove) sequence could not be presented because the giraffe left the area. These trials were retained for all analyses not directly involving responses to dove stimuli, given empirical evidence from previous work that giraffes do not exhibit behavioral reactions to dove calls (Baotic and Szipl 2025a, 2025b).

Playback amplitude was standardized as described above, while propagation trials were used to verify signal detectability and acoustic fidelity across field-relevant distances. This protocol ensured that behavioral responses could be attributed to the playback stimuli under controlled and ecologically relevant conditions.

### Behavioral data collection and coding

All behavioral responses during playback trials were recorded in high definition using a Sony FDR-AX53 video camera positioned to capture the focal giraffe throughout the trial. Videos were analyzed retrospectively with Solomon Coder (version 17.03), applying frame-by-frame scoring at a temporal resolution of 20 ms. Continuous sampling was used to accurately document both the onset and duration of each predefined behavior.

**Figure 1:**
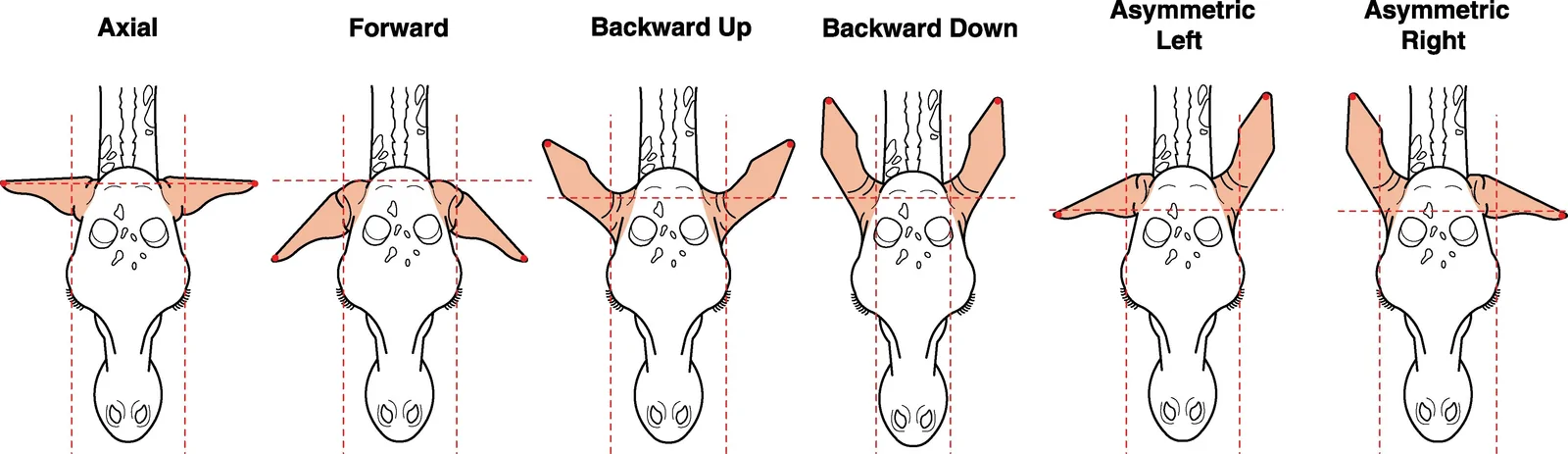
Ear posture categories employed to score giraffe vigilance behavior. Postures were defined based on ear orientation relative to the horizontal plane and the head-neck axis. Axial postures involve bilateral lateral ear extension. Forward postures are marked by anteriorly directed ears elevated more than 30°. Backward up and backward down postures describe posterior ear orientations positioned above or below the neck line, respectively. Asymmetric postures were recorded when ears differed in orientation between the left and right sides. Figure reproduced from Baotic and Szipl (2025b), BMC Biology, CC BY 4.0.

The ethogram applied in this study was based on previously validated giraffe playback protocols. Behavioral coding was not conducted blind to playback treatment. The same observer applied the previously validated behavioral scoring criteria established in earlier giraffe playback studies, for which high inter-observer reliability had been confirmed (Baotic and Szipl 2025a, 2025b). Behavioral variables consisted primarily of clearly observable duration-based behaviors and predefined behavioral categories, reducing ambiguity during scoring. Behavioral categories included foraging behaviors (“feeding” and “not feeding”), primary vigilance responses (“scanning”, “approach-investigation”, and “displacement”), as well as ear postures (“axial”, “forward”, “backward up”, “backward down”, and “asymmetric left/right”, Fig. 1), which were included as indicators of attentional orientation and vigilance state. “Feeding” was defined as continuous mouth-to-vegetation contact, while “not feeding” indicated a clear cessation of foraging activity. Vigilance was further characterized by the initial head and neck movement toward the sound source and stationary scanning with an elevated head position, cautious movement toward the loudspeaker (“approach-investigation”), and sustained walking away from the speaker (“displacement”). Behavioral categories were based on a previously established and validated ethogram (Baotic and Szipl 2025a, 2025b) and applied here using the criteria summarized in Table 1.

**Table 1:** Definitions of behavioral categories used to quantify giraffe responses to playback stimuli.

Behavior	Definition
**Feeding**	Continuous foraging behavior involving active mouth contact with leaves or branches, maintained for a minimum duration of 10 s.
**Not feeding**	Complete cessation of foraging activity, with no further interaction with vegetation.
**Ear posture**	Ear orientation classified into 5 discrete categories: axial, forward, backward up, backward down, and asymmetric left/right—used as indicators of attentional state.
**Scanning**	Active head movements associated with visual monitoring of the surroundings; repeated reorientation toward the loudspeaker is included in this category.
**Approach-Investigation**	Cautious locomotion toward the sound source, either directly or along curved trajectories, accompanied by sustained vigilance. This behavior requires a minimum of 3 steps toward the loudspeaker and may result in a stationary attentive posture, in which case scanning is scored.
**Displacement**	Sustained walking for at least 10 seconds away from the loudspeaker, indicating movement away from the stimulus source.

For each playback trial, the duration and latency to first occurrence were recorded for all scored behaviors. Among these, the latency from stimulus onset to the first occurrence of “not feeding” was used as the primary measure of rapid vigilance response, consistent with established protocols in previous giraffe playback studies. When a giraffe continued feeding throughout the 20-s post-stimulus observation window, no reaction occurred within the observation period. These trials were therefore treated as right-censored observations, reflecting biologically meaningful nonresponses rather than missing data, following established applications of survival analysis in animal behavior research (Jahn-Eimermacher et al. 2011; Asher et al. 2017).

### Statistical analysis

#### Response rate and dataset selection

To evaluate the effectiveness of the playback stimuli and confirm the suitability of the dove call as a neutral control, the proportion of trials in which giraffes responded to each stimulus type was first quantified. A response was defined as any instance where the “Total response time (s)” exceeded zero. For each playback category (lion, oxpecker, dove), the percentage of trials with a response was calculated by dividing the number of response trials by the total number of trials for that stimulus and multiplying by 100. Among the 35 giraffes tested, only one individual responded to the dove (control) playback, most likely reflecting residual vigilance from the preceding oxpecker alarm call, whereas 34 giraffes responded to the oxpecker alarm and all 35 responded to the lion playback. This corresponded to response rates of 2.9% for dove trials, 94.3% for oxpecker trials, and 100% for lion trials. The extremely low response rate to dove playbacks is consistent with previous playback experiments that dove coos function as robust neutral controls (Baotic and Szipl 2025a, 2025b; Taylor et al. 2025). As dove calls elicited no detectable vigilance response and functioned as a neutral stimulus, they were not considered to contribute meaningful risk information. Accordingly, sequence effects were interpreted based on the most recent biologically relevant stimulus (lion or oxpecker), irrespective of any intervening control playback.

#### Vigilance response (principal component analysis)

Behavioral responses were summarized using principal component analysis (PCA) to reduce redundancy among correlated vigilance behaviors and derive a continuous composite measure of overall vigilance responses (Budaev 2010).

Several vigilance-related behaviors could occur simultaneously (eg scanning while not feeding), meaning that total response time represented the overall duration of the vigilance episode rather than the sum of individual behavioral durations.

To quantify overall vigilance, the PCA was based on 4 biologically relevant duration measures: total response time, duration of not feeding, duration of ears forward, and duration of scanning behavior. These behaviors were selected because together they represent complementary components of antipredator vigilance in giraffes, encompassing interruption of foraging, attentional orientation, and sustained environmental monitoring (Baotic and Szipl 2025a, 2025b; Taylor et al. 2025). The resulting PCA produced a robust 1-component solution (KMO = 0.759; Bartlett's test: χ^2^ = 998.699, df = 6, *P* < 0.001), with all 4 variables loading strongly onto a single principal component (PC), which explained 95.4% of the total variance in behavioral responses (Table 2). Because these variables collectively reflected interruption of foraging and sustained attentional vigilance, the retained PC was interpreted as a composite vigilance-response axis and used in all subsequent analyses. Details of an exploratory PCA including all recorded behavioral variables are provided in Methods S3 and Table S1.

**Table 2:** Principal component loadings and descriptive statistics for vigilance-related behavioral durations.

Behavioral categories	Behavioral response duration (s)			PCA		
	Minimum (s)	Maximum (s)	Mean ± SD (s)	PC	*h* ^2^	*u* ^2^
Total response time	6.2	882.5	100.7 ± 139.2	0.987	0.975	0.025
Not feeding	0.0	492.9	79.4 ± 108.5	0.941	0.886	0.114
Ears forward	0.0	557.2	79.8 ± 113.6	0.996	0.992	0.008
Scanning	0.0	857.2	87.0 ± 131.2	0.981	0.962	0.038

PC extracted from PCA showing standardized loadings (PC), communalities (*h*^2^), and uniqueness (*u*^2^) for behavioral response durations (in seconds) measured during playback experiments. Sampling adequacy was confirmed by a Kaiser–Meyer–Olkin (KMO) measure of 0.759 and a significant Bartlett's test of sphericity (χ^2^ = 998.699, df = 6, *P* < 0.001).

#### General modeling strategy

Vigilance responses (PC) were analyzed using 3 complementary modeling approaches that addressed successive biological questions. The first model compared responses across all playback categories, including the neutral dove control. The second was restricted to lion and oxpecker playbacks to directly compare biologically relevant risk cues. The third model examined whether responses to the second playback within reciprocal lion-oxpecker sequences depended on the preceding behavioral response.

PC scores were analyzed using mixed-effects models with giraffe identity as a random intercept where repeated observations occurred. Candidate models reflected biologically motivated hypotheses. Models were fitted using maximum likelihood estimation to allow direct comparison of candidate models using Akaike's Information Criterion corrected for small sample size (AICc). Model assumptions were evaluated using *DHARMa*-based diagnostic procedures including assessments of dispersion, normality, outliers, residuals, and variance inflation factors (VIF). Complete candidate model sets and AICc comparison tables are provided in Tables S3, S5, and S6. For the best-supported model within each modeling framework, estimated marginal means (EMMs) were calculated using the *emmeans* package (Lenth 2025) to facilitate interpretation of significant fixed effects. Pairwise contrasts among factor levels were performed where appropriate, with *P*-values adjusted for multiple testing using Tukey's method.

#### Overall playback response model

The overall playback response model evaluated whether giraffe vigilance responses differed among lion, oxpecker, and dove playback categories while accounting for repeated observations and biologically relevant covariates. Dove playbacks were retained as a neutral control to assess whether responses to lion and oxpecker stimuli exceeded generalized reactions to nonthreatening acoustic stimuli.

Candidate linear mixed models (LMM) included stimulus type, playback order, sex, and playback distance as fixed effects, with giraffe identity included as a random intercept. Playback order was included because sequential presentation of stimuli could influence vigilance response independently of stimulus identity. Interaction models evaluating stimulus type × playback order and stimulus type × sex were also considered. Additional robustness analyses incorporating playback date and playback stimulus identity as random effects are also provided in Table S3.

#### Lion–oxpecker playback subset model

To directly compare responses to biologically relevant risk cues, a second model framework was restricted to lion and oxpecker playback stimuli. Dove playbacks were excluded because they served as neutral control stimuli and were not directly relevant to hypotheses concerning predator cue weighting or heterospecific alarm processing. Candidate LMMs retained the same fixed- and random-effects structure as the overall playback response model, with the addition of a biologically motivated stimulus type × sex interaction to test whether cue-specific vigilance responses differed between males and females. Model sets are provided in Table S5.

#### Preceding playback context model

To evaluate whether vigilance responses to a playback were influenced by the preceding playback, analyses were restricted to reciprocal lion-oxpecker playback combinations (ie, lion first–oxpecker second; oxpecker first–lion second). Dove playbacks were excluded because they served as neutral control stimuli and were not relevant to hypotheses concerning preceding playback context. The model therefore tested whether responses to the second playback stimulus covaried with the magnitude of the preceding vigilance response.

The response variable was the vigilance score of the second playback stimulus (PC 2^nd^ stimulus). Candidate models included second-stimulus identity, the magnitude of the preceding vigilance response (PC 1^st^ stimulus), sex, and inter-stimulus interval duration as explanatory variables. Because first- and second-stimulus identities were structurally linked within the reciprocal playback design, only second-stimulus identity was included in the models. Interaction terms between second-stimulus identity and preceding vigilance response were additionally evaluated. Because inter-stimulus intervals depended on natural field conditions and continuous visibility of focal individuals, interval duration was included as a covariate rather than experimentally controlled. The dataset was reformatted so that each sequential playback combination contributed a single observation consisting of the vigilance response to the second playback (PC 2^nd^ stimulus), together with the corresponding preceding vigilance response (PC 1st stimulus), second-stimulus identity, sex, and inter-stimulus interval. Because the reformatted dataset no longer contained repeated observations requiring a random effect, standard linear models were fitted. Candidate models were compared using AICc, with full model sets provided in Table S6.

#### Latency to “Not Feeding”

To further characterize the temporal dynamics of behavioral responses, latency to “Not Feeding” (ie, time until cessation of feeding following playback onset) was analyzed as a time-to-event variable. The event was defined as the first transition from “feeding” to “not feeding”. Trials in which individuals continued feeding throughout the observation period were treated as right-censored at 20 s, corresponding to the maximum latency observation window. The 20-s window applied only to the latency analysis of feeding cessation and did not represent the total duration of behavioral observation or the interval between successive playback stimuli.

To account for nonindependence arising from repeated playback presentations to the same individuals, shared frailty Cox proportional hazards models were fitted with giraffe identity included as a shared frailty term. Models were fitted using the R package *frailtyHL* assuming a log-normal frailty distribution and using the HL(1,1) estimation procedure (Do Ha et al. 2012). Stimulus type, playback order, and sex were included as fixed effects, corresponding to the best-supported fixed-effect structure identified in the lion-oxpecker subset model. To evaluate the contribution of giraffe-level frailty, the frailty model was additionally compared with a corresponding Cox model in which the frailty variance was fixed to zero, allowing assessment of whether including giraffe identity improved model fit.

#### Statistical software

Statistical analyses were performed in R Studio Version 2024.12.1+563 (RStudio Team 2025) using R Version 4.5.0 (R Core Team 2025). PC analyses were conducted using the package *psych* (Revelle 2024). Linear-mixed models assuming Gaussian error distributions with identity link functions were fitted using the *lme4* package (Bates et al. 2015). Estimated marginal means (EMMs) and pairwise contrasts were calculated using the *emmeans* package (Lenth 2025). Latency analyses were performed using the packages *survival* (Therneau 2024) and *frailtyHL* (Do et al. 2019), and model diagnostics were evaluated using *DHARMa* (Hartig 2024). The complete source datasets and representative videos of the playback trials are publicly available in the Zenodo repository (Baotic 2026).

## Results

### Overall playback response model

The best-supported overall playback response model, selected from 8 candidate models including a null model, included stimulus type, playback order, sex, and playback distance as fixed effects, with giraffe identity included as a random intercept (Table S3). This additive model received the strongest AICc support (AICc = 250.15), although a model including a stimulus type × sex interaction also received substantial support (ΔAICc = 0.36). The additive model was retained as the primary model because it provided the most parsimonious explanation of variation in vigilance responses and showed low multicollinearity among predictors.

Playback responses differed significantly among stimulus categories (Table 3). Compared with dove control calls, giraffes showed substantially stronger vigilance responses to both lion roars (estimate = 1.423 ± 0.182 SE, 95% CI = 1.066 to 1.780, *P* < 0.001) and oxpecker alarm calls (estimate = 0.711 ± 0.187 SE, 95% CI = 0.345 to 1.077, *P* < 0.001), whereas lion roars elicited the strongest responses. EMMs and Tukey-adjusted pairwise comparisons (Table 3) similarly indicated highest vigilance responses during lion playback trials, intermediate responses during oxpecker alarm-call trials, and lowest responses during dove playback trials.

**Table 3:** Summary of the overall linear mixed model (LMM) testing the effects of playback stimulus type, playback order, sex, and playback distance on giraffe vigilance response scores.

Panel A. Fixed effects from the overall LMM					
Fixed effects	Estimate	SE	95% CI	*t*	*P*
Intercept (Dove, Female, Order 1)	−0.093	0.312	−0.705 to 0.520	−0.297	0.767
Lion relative to Dove	1.423	0.182	1.066 to 1.780	7.81	<0.001
Oxpecker relative to Dove	0.711	0.187	0.345 to 1.077	3.81	<0.001
Order 2 relative to Order 1	−0.016	0.177	−0.364 to 0.331	−0.090	0.928
Order 3 relative to Order 1	0.438	0.187	0.072 to 0.805	2.345	0.022
Male relative to Female	−0.479	0.155	−0.783 to −0.175	−3.090	0.004
Playback distance	−0.0068	0.0031	−0.0128 to −0.0008	−2.206	0.032

Panel B. Estimated marginal means (EMMs)				
Estimated marginal means	EMM	SE	df	95% CI
Dove playback	−0.696	0.139	110	−0.972 to −0.420
Lion playback	0.727	0.131	109	0.467 to 0.987
Oxpecker playback	0.015	0.134	110	−0.250 to 0.281
Order 1	−0.125	0.133	110	−0.389 to 0.138
Order 2	−0.141	0.132	110	−0.403 to 0.120
Order 3	0.313	0.141	110	0.034 to 0.592
Female	0.255	0.12	39.9	0.011 to 0.498
Male	−0.224	0.106	36.5	−0.440 to −0.009

Panel C. Pairwise contrasts					
Pairwise contrasts	Estimate	SE	df	t	*P*
Dove vs Lion	−1.423	0.188	73.3	−7.561	<0.001
Dove vs Oxpecker	−0.711	0.193	72.9	−3.69	<0.001
Lion vs Oxpecker	0.711	0.185	71.6	3.846	<0.001
Order 1 vs Order 2	0.016	0.183	71.5	0.088	0.931
Order 1 vs Order 3	−0.438	0.193	74.2	−2.27	0.039
Order 2 vs Order 3	−0.454	0.191	73.9	−2.376	0.039

Fixed-effect estimates are shown relative to the reference categories Dove playback, Female, and Order 1. Estimated marginal means (EMMs) and pairwise contrasts were derived from the fitted model. Confidence intervals are shown as 95% CIs. Pairwise comparison *P* values were Tukey-adjusted.

The additive model further indicated increased vigilance responses during the third playback order across stimulus categories (Table 3). Tukey-adjusted post hoc comparisons revealed that responses during playback order 3 were significantly stronger than responses during playback orders 1 and 2, whereas responses did not differ between playback orders 1 and 2. Interpretation of this order effect should nevertheless remain cautious because playback order was partially confounded with stimulus composition, as lion playbacks occurred more frequently than oxpecker alarm calls in the third playback position (Table S2). Although interaction models between stimulus type and playback order were additionally evaluated, they provided limited additional explanatory value and showed substantially higher collinearity among predictors (for a tabular overview see Table S3).

Male giraffes additionally showed lower vigilance responses than females (estimate = −0.479 ± 0.155 SE, 95% CI = −0.783 to −0.175, *P* = 0.004; Table 3). Vigilance responses also showed a modest negative association with playback distance (estimate = −0.007 ± 0.0031 SE, 95% CI = −0.013 to −0.001, *P* = 0.032; Table 3). However, playback distances did not differ among stimulus categories (Kruskal–Wallis: χ^2^ = 0.60, df = 2, *P* = 0.741). Additionally, females tended to be tested at slightly greater distances than males (Table S4), although this difference was not statistically significant (Wilcoxon–Mann–Whitney *U* test: W = 1599.5, *P* = 0.056). The model explained a substantial proportion of variance in vigilance response (marginal *R*^2^ = 0.438; conditional *R*^2^ = 0.449), whereas individual identity contributed comparatively little additional variance (ICC = 0.020). DHARMa model diagnostics indicated no evidence of over dispersion (dispersion test: dispersion = 1.007, *P* = 0.896) or problematic outliers (outlier test: *P* = 0.185). Residual diagnostics nevertheless revealed a modest but significant deviation from complete residual uniformity (Kolmogorov–Smirnov test: D = 0.161, *P* = 0.010), suggesting some remaining residual structure within the behavioral dataset.

### Lion–oxpecker playback subset model

The best-supported lion-oxpecker response model included stimulus type, playback order, sex, and playback distance as fixed effects, with giraffe identity included as a random intercept. This additive model received the strongest AICc support (AICc = 198.4, AICc weight = 0.73), whereas the interaction model including a stimulus type × sex interaction received weaker support (ΔAICc = 2.03, AICc weight = 0.27) and did not improve interpretation of the response patterns. The additive model was therefore retained as the primary model because the interaction term was not statistically supported and did not sufficiently improve model fit relative to the increase in model complexity (Table S5). Within the lion-oxpecker playback subset, giraffes responded significantly more strongly to lion roars than to oxpecker alarm calls (oxpecker relative to lion: estimate = −0.737 ± 0.204 SE, 95% CI = −1.138 to −0.337, *P* < 0.001; Table 4). EMMs similarly indicated substantially stronger vigilance responses during lion playback trials (EMM ± SE: 0.719 ± 0.158, 95% CI = 0.403 to 1.034) compared with oxpecker alarm-call trials (−0.019 ± 0.158, 95% CI = −0.334 to 0.296).

**Table 4:** Results of the best-supported linear mixed subset model examining giraffe vigilance responses to lion roars and oxpecker alarm calls.

Panel A. Fixed effects from the lion-oxpecker subset mixed model					
Fixed effects	Estimate	SE	95% CI	*t*	*P*
Intercept (Lion, Female, Order 1)	1.484	0.415	0.671 to 2.297	3.578	<0.001
Oxpecker relative to Lion	−0.737	0.204	−1.138 to −0.337	−3.611	<0.001
Playback order (Order 2 relative to Order 1)	0.401	0.205	−0.0003 to 0.802	1.959	0.058
Male relative to Female	−0.649	0.229	−1.097 to −0.200	−2.837	0.008
Playback distance	−0.009	0.005	−0.018 to 0.0001	−1.931	0.06

Panel B. Estimated marginal means (EMMs)				
Estimated marginal means	EMM	SE	df	95% CI
Lion playback	0.719	0.158	74.8	0.403 to 1.034
Oxpecker playback	−0.019	0.158	74.8	−0.334 to 0.296
Female	0.674	0.175	38.2	0.320 to 1.030
Male	0.025	0.16	38.2	−0.299 to 0.350

Fixed-effect estimates are shown relative to the reference categories Lion playback, Female, and Order 1. Estimated marginal means were derived from the fitted model using Kenward–Roger degrees of freedom. Confidence intervals are 95% CIs.

**Figure 2:**
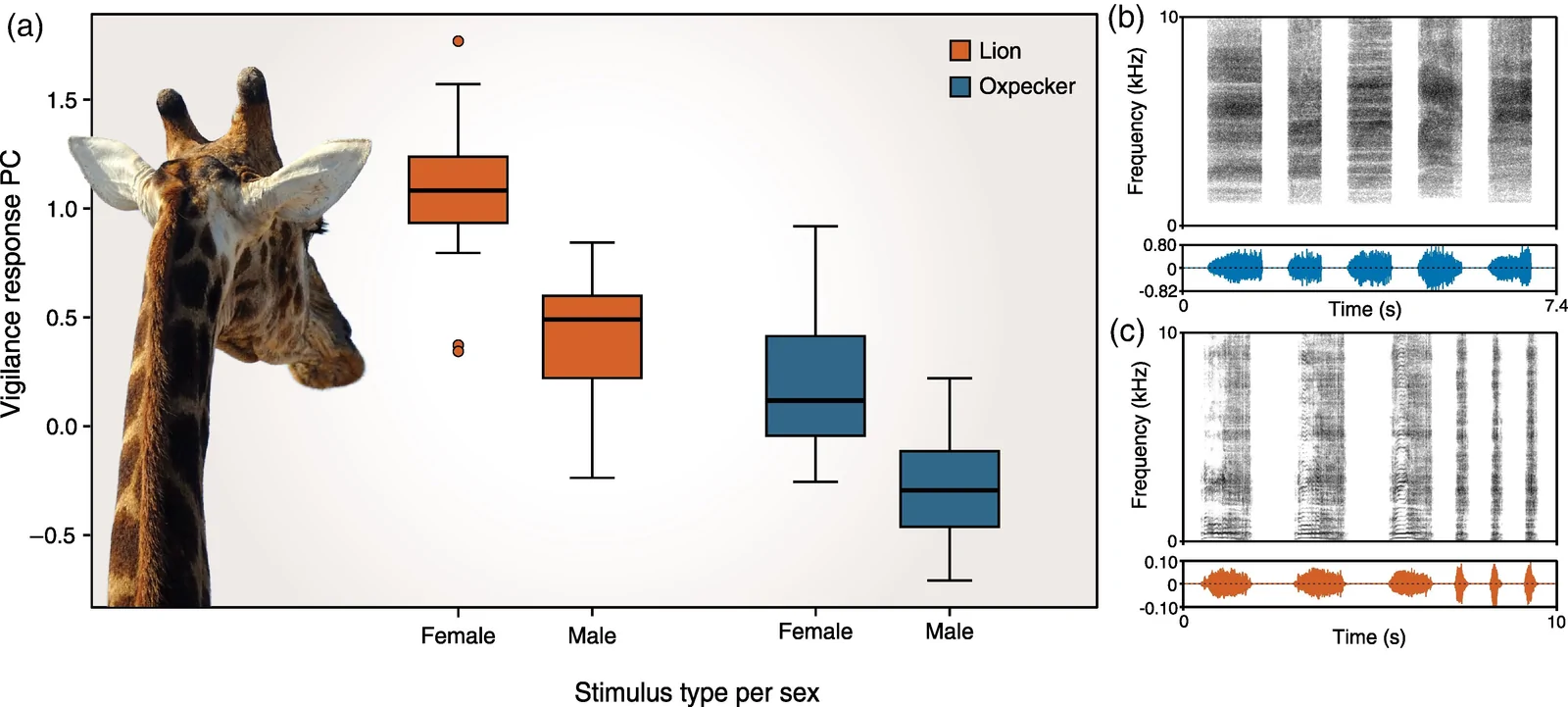
Vigilance responses of female and male giraffes to lion roars and oxpecker alarm calls. a) Distribution of vigilance-response PC scores representing an integrated vigilance response axis based on interruption of feeding, scanning behavior, ears-forward orientation, and total vigilance duration. Giraffes exhibited stronger vigilance responses to lion roars than to oxpecker alarm calls, while females generally showed higher vigilance-response scores than males. b) Spectrogram (top) and waveform (bottom) of a red-billed oxpecker ‘ksss’ alarm call sequence used for playback. c) Representative spectrogram (top) and waveform (bottom) of a lion roar-grunt sequence used for playback, consisting of 3 full roars followed by 3 grunts (recorded by Timmy Moser). Spectrograms were generated in Praat using a fast Fourier transform (FFT) with a dynamic range of 50 dB. Window length was set to 0.04 s for the oxpecker stimulus and 0.07 s for the lion stimulus.

Male giraffes additionally showed significantly lower vigilance response scores than females (estimate = −0.649 ± 0.229 SE, 95% CI = −1.097 to −0.200, *P* = 0.008; Table 4). EMMs further indicated higher vigilance responses in females (0.674 ± 0.175, 95% CI = 0.320 to 1.030) than males (0.025 ± 0.160, 95% CI = −0.299 to 0.350) (Table 4, Fig. 2). However, the interaction between stimulus type and sex received comparatively weak support and was not statistically significant (Table S5), indicating that both sexes showed broadly similar discrimination patterns between lion roars and oxpecker alarm calls despite differing in their overall vigilance response. Playback order showed a weak positive trend but did not significantly influence vigilance response magnitude (estimate = 0.401 ± 0.205 SE, 95% CI = −0.0003 to 0.802, *P* = 0.058). Similarly, playback distance showed no clear effect on vigilance response within the lion-oxpecker subset (estimate = −0.009 ± 0.005 SE, 95% CI = −0.018 to 0.0001, *P* = 0.060) (Table 4).

The model explained a moderate proportion of variance in vigilance response (marginal *R*^2^ = 0.283; conditional *R*^2^ = 0.361), with individual identity accounting for 10.8% of the total variance in vigilance responses (ICC = 0.108). *DHARMa* simulation-based diagnostics did not indicate significant violations of model assumptions. Residual uniformity tests were nonsignificant overall (Kolmogorov–Smirnov test: *D* = 0.127, *P* = 0.207), and no evidence for over dispersion (*P* = 0.870) or problematic outliers (*P* = 0.131) was detected.

### Preceding playback context model

This analysis evaluated whether vigilance responses to successive lion-oxpecker playback sequences were influenced by preceding playback context across inter-stimulus intervals averaging 120.3 ± 66.9 s (range: 29.9 to 270.9 s). The additive model including second-stimulus identity, preceding vigilance response, and sex was identified as the best-supported model (AICc = 107.21, AICc weight = 0.52; Table S5). Models additionally including inter-stimulus interval duration (ΔAICc = 0.77, AICc weight = 0.36) or biologically motivated interaction terms between second-stimulus identity and preceding response magnitude (ΔAICc = 3.82, AICc weight = 0.08) received comparatively weaker support. The additive model was therefore retained as the primary model because it provided the best balance between explanatory power and model complexity (Table S6).

Within the best-supported model (Table 5), males exhibited significantly lower vigilance responses to the second playback stimulus than females (estimate = −0.896 ± 0.365 SE, 95% CI = −1.640 to −0.152, *t* = −2.456, *P* = 0.020; Table 5). EMMs similarly indicated higher vigilance responses in females (0.999 ± 0.270, 95% CI = 0.449 to 1.549) than males (0.103 ± 0.232, 95% CI = −0.370 to 0.576; Table S6).

**Table 5:** Results of the best-supported preceding playback context response linear model, evaluating whether vigilance responses to the second playback stimulus were associated with second-stimulus identity, preceding response magnitude, and sex.

Panel A. Fixed effects from the preceding playback context linear model					
Fixed effects	Estimate	SE	95% CI	*t*	*P*
Intercept (Second lion stimulus, Female)	1.29	0.267	0.747 to 1.834	4.841	<0.001
Second oxpecker relative to second lion stimulus	−0.620	0.393	−1.421 to 0.181	−1.578	0.125
Preceding vigilance response (PC 1^st^ stimulus)	0.21	0.2	−0.198 to 0.617	1.049	0.302
Male relative to Female	−0.896	0.365	−1.640 to −0.152	−2.456	0.02

Panel B. Estimated marginal means (EMMs)				
Estimated marginal means	EMM	SE	df	95% CI
Second lion playback	0.861	0.235	31	0.381 to 1.340
Second oxpecker playback	0.241	0.286	31	−0.334 to 0.824
Female	0.999	0.270	31	0.449 to 1.549
Male	0.103	0.232	31	−0.370 to 0.576

Fixed-effect estimates are shown relative to the reference categories second lion stimulus and female giraffes. Estimated marginal means (EMMs) were derived from the fitted model. Confidence intervals are shown as 95% CIs.

Responses remained lower when oxpecker alarm calls were presented as the second playback stimulus compared with second lion playbacks, although this effect did not reach statistical significance (estimate = −0.620 ± 0.393 SE, 95% CI = −1.421 to 0.181, *t* = −1.578, *P* = 0.125; Table 5). EMMs nevertheless indicated stronger vigilance responses during second lion playback trials (0.861 ± 0.235, 95% CI = 0.381 to 1.340) compared with second oxpecker playback trials (0.241 ± 0.286, 95% CI = −0.343 to 0.824).

In contrast, the magnitude of the preceding behavioral response was not significantly associated with responses to the second playback stimulus (estimate = 0.210 ± 0.200 SE, 95% CI = −0.198 to 0.617, *t* = 1.049, *P* = 0.302; Table 5), indicating no clear evidence that stronger initial reactions predicted stronger subsequent vigilance responses across playback sequences. Likewise, inclusion of inter-stimulus interval duration slightly increased explained variance but did not improve overall model support based on AICc, and interval duration itself was not a significant predictor of second-stimulus responses (estimate = −0.004 ± 0.003 SE, 95% CI = −0.010 to 0.002, *t* = −1.383, *P* = 0.177; Table S7). Interaction models additionally indicated no evidence that relationships between preceding and subsequent responses differed according to second-stimulus identity (interaction term: *P* = 0.775; Table S8).

The best-supported preceding playback context model explained a moderate proportion of variance in second-stimulus responses (*R*^2^ = 0.302; adjusted *R*^2^ = 0.234). *DHARMa* residual diagnostics indicated no evidence of residual nonuniformity (Kolmogorov–Smirnov test: *D* = 0.171, *P* = 0.228), dispersion problems (dispersion = 0.911, *P* = 0.776), problematic outliers (*P* = 1.000), or systematic residual structure across fitted values and predictors.

### Latency to “Not Feeding”

Mixed-effects survival analysis indicated no significant effects of stimulus type, playback order, or sex on latency to interrupt feeding. In the shared frailty Cox proportional hazards model, giraffes showed no significant difference in latency responses between lion and oxpecker playback conditions (oxpecker relative to lion: estimate = −0.297 ± 0.266 SE, *t* = −1.117, *P* = 0.264). Playback order similarly showed no detectable effect on latency to interrupt feeding (second relative to first playback stimulus: estimate = −0.103 ± 0.263 SE, *t* = −0.393, *P* = 0.694), and sex also did not significantly influence latency responses (males relative to females: estimate = −0.197 ± 0.283 SE, *t* = −0.697, *P* = 0.486). The estimated giraffe-level frailty variance was low (0.132 ± 0.205 SE). Comparison with a corresponding Cox model in which frailty variance was fixed to zero indicated little evidence that including giraffe identity improved model fit, as restricted likelihood deviance values differed only marginally between models (456.10 vs. 456.68; Δ = 0.18). Relative model support was similarly comparable between models (rAIC = 458.10 vs. 456.68) (see Table S9).

## Discussion

The present study demonstrates that giraffe vigilance responses varied according to the informational content and perceived relevance of different acoustic risk cues. Across analyses, lion vocalizations consistently elicited the strongest vigilance responses regardless of playback position, indicating prioritization of direct predator-associated information over indirect heterospecific cues. In contrast, responses to oxpecker alarm calls were weaker and more variable across playback conditions, suggesting that giraffes weighted these indirect alarm cues less strongly than direct predator vocalizations. Females also tended to exhibit stronger vigilance responses than males in several analyses, indicating possible sex-related differences in antipredator investment. Evidence that preceding playback context strongly altered subsequent vigilance responses was limited, as giraffes continued to discriminate between cue types despite prior exposure. Although giraffes rapidly interrupted feeding following both lion and oxpecker playbacks, differences between cue types were more apparent in the magnitude of vigilance behavior than in response latency. Together, these findings suggest that giraffes differentially weight multiple sources of risk-related information according to their relative salience and ecological significance.

### Differential weighting of lion and oxpecker calls

The consistently strong responses to lion vocalizations indicate that giraffes treated direct predator cues as highly salient indicators of immediate danger. Lion roars elicited stronger vigilance responses than oxpecker alarm calls regardless of playback order, suggesting that predator-associated information retained high behavioral importance even when preceded by other potentially threatening stimuli. Such a pattern is broadly consistent with theoretical expectations that prey should prioritize cues associated with comparatively high reliability and immediate fitness consequences when assessing predation risk (McNamara and Houston 1986; Lima and Dill 1990; Dall et al. 2005). More broadly, African herbivores have previously been shown to adjust antipredator behavior according to predator identity and perceived lethality. For example, larger-bodied ungulates, including giraffes, preferentially avoided areas characterized by high lion and leopard activity, suggesting selective behavioral responses toward predators posing comparatively greater risk (Thaker et al. 2011). Experimental playback studies further demonstrate that lion vocalizations can rapidly increase vigilance and defensive grouping behavior in African herbivores (Dannock et al. 2019), while Rigoudy et al. (2022) showed that African ungulates respond differentially to predator vocalizations according to perceived predator lethality.

Although lion roars primarily function in long-distance communication, territorial advertisement, and social coordination rather than active stalking behavior (Schaller 1976; Grinnell and McComb 2001), they nevertheless provide reliable information about predator presence and elevated local risk within the environment. Lions represent one of the principal predators of giraffes, particularly calves but also occasionally vulnerable adults under specific ecological conditions (Hayward and Kerley 2005; Valeix et al. 2009a; Périquet et al. 2010; Bennitt et al. 2024). Numerous prey species respond strongly to predator vocalizations despite the absence of visual predator confirmation, including African savanna elephants (*Loxodonta africana*) exposed to lion calls (McComb et al. 2011) and humpback whales (*Megaptera novaeangliae*) to killer whale (*Orcinus orca*) sounds, exposed to predator-associated acoustic cues (Curé et al. 2015). The extremely limited responses to dove control calls further indicate that giraffes discriminated selectively among playback categories rather than responding indiscriminately to novel acoustic stimuli.

Despite weaker overall responses relative to lion vocalizations, giraffes nevertheless responded consistently to oxpecker alarm calls, indicating that these heterospecific vocalizations conveyed ecologically relevant information. Animals frequently exploit heterospecific-derived information about predators, particularly when species differ in vigilance, detection ability, or access to environmental information (Magrath et al. 2015; Goodale and Ruxton 2019). Such responses do not necessarily require close social relationships between species. For example, Lea et al. (2008) demonstrated that Gunther's dik-diks (*Madoqua guentheri*), a nonsocial and highly predator-vulnerable antelope species, responded strongly to alarm calls of white-bellied go-away birds despite possessing relatively simple vocal communication themselves. The authors argued that elevated predation risk alone may favor the evolution of heterospecific eavesdropping in vulnerable prey species (Lea et al. 2008).

A similar mechanism may contribute to giraffe responsiveness toward oxpecker vocalizations. Oxpeckers are highly vigilant birds that occupy elevated positions on large mammals and frequently produce sharp hissing-like “ksss” calls during disturbance and vigilance contexts (Atwell 1966; Maclean et al. 1988). Mammalian hosts have previously been shown to attend to oxpecker vocal behavior; for example, black rhinoceroses respond to oxpecker alarm calls during human approaches, supporting the idea that hosts can exploit oxpecker vocalizations through heterospecific eavesdropping rather than direct social communication (Plotz and Linklater 2020). Similarly, previous playback experiments demonstrated that giraffes respond robustly to oxpecker alarm calls even in the absence of oxpeckers themselves (Baotic and Szipl 2025b). The present findings extend this work by showing that, although oxpecker calls are meaningful risk-related cues for giraffes, they are weighted less strongly than direct predator-associated signals when both cue types are compared within the same experimental framework.

The comparatively weaker and more variable responses to oxpecker calls suggest that giraffes assigned less behavioral weight to these indirect heterospecific cues than to direct predator vocalizations. Because oxpecker alarm calls may occur across a broader range of disturbance contexts, maintaining responsiveness to such cues while avoiding excessive vigilance may therefore allow giraffes to benefit from early-warning information without incurring unnecessary foraging disruption (Lima and Dill 1990; Preisser et al. 2005). Together, these findings suggest that giraffes differentially weighted multiple sources of risk-related information according to their immediacy, reliability, and ecological relevance.

### Effects of preceding playback context across successive playbacks

Evidence that preceding playback context strongly influenced subsequent vigilance responses was limited. Responses to lion vocalizations remained consistently strong regardless of preceding playback condition, while the magnitude of preceding vigilance responses showed little association with subsequent responses. Together, these findings indicate that differences in vigilance responses to lion vocalizations and oxpecker alarm calls remained broadly stable across successive playbacks rather than reflecting generalized carry-over effects. Previous studies have shown that predator-associated acoustic cues can induce prolonged behavioral changes that persist beyond the immediate exposure period (Abbey-Lee et al. 2016; Santema et al. 2019). Similarly, elevated responses following alarm cues may sometimes reflect transient agitation or short-term arousal rather than sustained integration of prior information (McIvor et al. 2018). Because inter-stimulus intervals were not experimentally standardized under field conditions, the present design cannot completely exclude some contribution of residual arousal from the preceding playback stimulus. Nevertheless, giraffes consistently responded more strongly to lion vocalizations than to oxpecker alarm calls regardless of playback order, suggesting that responses were driven primarily by the identity of the current playback cue rather than by generalized carry-over effects under the conditions examined.

### Sex difference in vigilance responses

Females generally exhibited stronger vigilance responses than males across several analyses, suggesting sex-related variation in overall antipredator responsiveness or risk sensitivity. However, the absence of a statistically supported stimulus type × sex interaction indicates that males and females showed broadly similar relative responses to lion vocalizations and oxpecker alarm calls. Thus, sex appeared to influence overall vigilance responsiveness, whereas both sexes showed the same general pattern of responding more strongly to lion vocalizations than to oxpecker alarm calls.

Similar patterns have been reported across a range of ungulates, where females often maintain elevated vigilance under predation risk due to differences in reproductive investment, offspring vulnerability, or risk sensitivity (Hunter and Skinner 1998; Lashley et al. 2014; Pecorella et al. 2019). Male giraffes are often solitary, move extensively between female groups (Bercovitch et al. 2006), and are substantially larger-bodied than females (Cavener et al. 2024), potentially differing from females in both social environment and perceived vulnerability to predators. Lions preferentially target calves and more vulnerable individuals, whereas healthy adult bulls are comparatively less frequently preyed upon (Hayward and Kerley 2005), potentially contributing to weaker vigilance responses toward predator-associated cues.

At the same time, vigilance behavior in giraffes appears to be strongly influenced by social context. Cameron and du Toit (2005) found that cows scanned more frequently than bulls, although overall scan effort was broadly similar between sexes, and that vigilance varied according to the sex, age class, and proximity of nearby conspecifics. More recent studies further demonstrate that giraffe social associations are context dependent, particularly during foraging, and that social structure and association patterns differ between males and females (Bercovitch and Berry 2013; VanderWaal et al. 2013; Muller et al. 2018). Accordingly, local social context might also have contributed to the observed variation in vigilance responses. In the present study, males were typically tested while solitary, whereas females were generally tested while browsing within dispersed herds without nearby adult bulls. Although coordinated flight or strongly synchronized vigilance responses were not observed during playback trials, fine-scale social relationships and nearest-neighbor distances were not systematically quantified under field conditions and therefore were not included in the analysis. Consequently, some of the observed sex differences might partly reflect unmeasured variation in local social environment rather than sex alone. Future playback studies integrating detailed social-network information, nearest-neighbor identity and calf presence will help disentangle these potentially interacting effects.

### Latency

Notably, latency to stop feeding did not differ markedly across playback sequences, indicating that the timing of initial vigilance responses was relatively similar. In contrast, vigilance-response magnitude varied substantially across playback contexts, suggesting that cue type influenced the magnitude of behavioral responses more strongly than the timing of vigilance initiation. This pattern indicates that giraffes responded rapidly to both cue types, whereas the magnitude of the subsequent vigilance response varied according to cue type. Here, vigilance-response magnitude refers to overall vigilance investment quantified using a composite measure integrating multiple vigilance-related behaviors rather than differences among individual vigilance behaviors.

Interruptions of feeding represent a biologically meaningful cost in large herbivores, as vigilance-foraging tradeoffs directly constrain energy intake and are shaped by both predation risk and social factors (Freidly et al. 2025). The present findings therefore support previous work showing that antipredator responses may involve rapid initial detection followed by differences in behavioral investment according to perceived risk (Lima and Dill 1990; Stankowich and Blumstein 2005; McLachlan and Magrath 2020).

## Conclusion

The present study demonstrates that giraffes differentially respond to direct predator vocalizations and heterospecific alarm calls, with lion vocalizations consistently eliciting the strongest vigilance responses across playback contexts. In contrast, responses to oxpecker alarm calls were weaker and more variable, while preceding playback exposure showed limited influence on subsequent vigilance behavior. Females additionally exhibited generally stronger vigilance responses than males, suggesting that antipredator responsiveness may also vary according to sex-specific ecological and social factors. Together, these findings indicate that giraffes selectively weight different sources of risk-related information rather than responding uniformly to all potentially threatening acoustic cues. More broadly, the study contributes to growing evidence that large herbivores differentially weight direct predator-associated cues and heterospecific alarm cues when making antipredator decisions.

## Supplementary Material

arag099_Supplementary_Data

## Data Availability

Analyses reported in this article can be reproduced using the data provided by Baotic (2026).
