# Supplementary material for: Differential weighting of lion vocalizations and oxpecker alarm calls in giraffes
Source: Behav Ecol. 2026 Aug 20;37(5):arag099. doi: 10.1093/beheco/arag099 (PMC13526515; doi:10.1093/beheco/arag099)
Supplement: Supplementary file 1 [file arag099_supplementary_data.pdf]

Supplementary Material

Differential weighting of lion vocalizations and oxpecker alarm calls in giraffes

Anton Baotic[1*]

[1]Acoustics Research Institute, Austrian Academy of Sciences, Vienna, Austria

*** Correspondence:**
contact@antonbaotic.com

Supplementary Methods S1-S3:

Supplementary Methods S1. Playback stimuli:

Bird calls were recorded at 'Adventures with Elephants' (Limpopo, South Africa) between July and August 2023 using a Sound Devices MixPre-3 II recorder (frequency response: 10–80 kHz) and a Neumann KM183 MT omnidirectional microphone (frequency response: 10–20 kHz) fitted with a Rycote Cyclone windshield to minimize wind noise. Recordings were made in WAV format (44.1 kHz, 16-bit).

Additional red-billed oxpecker alarm calls were sourced from the Xeno-Canto archive: XC590464 (Elliott 1995), XC718396 (Rudman 2022a), XC718397 (Rudman 2022b), as well as Blue Pigeons Productions UK. Dove coo calls were sourced from XC810692 (Leite 2023a) and XC810974 (Leite 2023b), while lion calls were obtained from the Borror Laboratory of Bioacoustics sound archive (BLB25765, BLB25827, BLB25739) and collaborators (see Acknowledgements) to maximize stimulus diversity.

All recordings were visually inspected for quality using spectrograms in Praat 6.3.09 (Boersma and Weenink 2023), and only recordings with intact acoustic structure and minimal background interference were retained for playback preparation.
Ambient background noise was reduced in Audacity 3.3 (Audacity Team 2023) by applying the 'noise reduction' effect using one-second pre-call noise profiles (4–10 dB reduction, sensitivity 6.0, three smoothing bands), following approaches shown to preserve the spectral and temporal characteristics of animal vocalizations (Baker and Logue 2007; Benedict et al. 2021).

Species-specific spectral filtering was performed in Praat to remove frequencies outside the signal range of each playback category. Dove calls were filtered below 450 Hz and above 1.3 kHz. Lion vocalizations were filtered below 30 Hz and above 20 kHz. Oxpecker alarm calls were high-pass filtered below 720 Hz while all higher-frequency components were retained. All playback files were subsequently normalized to –1.0 dB peak amplitude to standardize playback level while avoiding clipping artefacts.

Playback amplitude was standardized to 85 dB SPL (Z-weighted) at 1 m. Because published source-level amplitude measurements are currently unavailable for red-billed oxpecker alarm calls, the selected playback level ensured clear signal detectability while remaining within the range reported for several similarly sized passerines and alarm-calling bird species.

Reported amplitudes include approximately 85 dB SPL in orchard orioles (*Icterus spurius*) (Moyer et al. 2024), 85–95 dB SPL in red-winged blackbirds (*Agelaius phoeniceus*) *(Brenowitz 1982)*, and 93–95 dB SPL for experimentally broadcast alarm calls of Grey go-away birds (*Crinifer concolor*) *(Lea et al. 2008)*. By comparison, vocalizations of Streptopelia doves are typically produced at lower amplitudes of approximately 75–80 dB SPL (den Hartog et al. 2008), whereas lion roars naturally reach higher levels, reported at approximately 114 dB SPL at 1 m (McComb et al. 1994).

Supplementary Methods S2. Playback design:

Twenty-eight playback packages were assembled before fieldwork. Each package comprised one lion, one oxpecker and one dove playback sequence presented in a predetermined order. Lion sequences consisted of three roar and three grunt calls selected from a pool of 84 roars (mean ± SD: 1.6 ± 0.4 s) and 84 grunts (0.6 ± 0.2 s) recorded from 15 individual lions. Dove sequences included six coo calls (from 138 calls, 1.06 ± 0.05 s), resulting in 23 unique dove sequences (7.5 ± 0.3 s). Oxpecker alarm sequences consisted of five alarm calls selected from a pool of 120 calls (0.9 ± 0.3 s), resulting in 24 unique sequences (8.0 ± 1.7 s), with seven oxpecker sequences reused to complete the full set of playback packages.

Because 35 giraffes were ultimately tested whereas only 28 playback packages had been prepared, 21 playback packages were used once and seven were used twice. No playback package was presented to more than two focal individuals.

Supplementary Methods S3. Exploratory PCA:

An exploratory PCA including all recorded behavioral variables was conducted to examine the overall covariance structure of the behavioral dataset. Although variables were significantly intercorrelated (Bartlett's test: $\chi^2 = 1891.578$, df = 45, $p < 0.001$), sampling adequacy was comparatively low (KMO = 0.529), and locomotor behaviors together with infrequently expressed ear-posture variables loaded on components separate from the primary vigilance axis (Supplementary Table S1). These results indicated that the complete behavioral dataset represented multiple behavioral dimensions rather than a single underlying vigilance construct. Accordingly, the main analyses used a PCA based on four biologically relevant vigilance-related duration measures (total response time, duration of not feeding, duration of ears forward, and duration of scanning behavior), which produced the robust one-component solution described in the main text.

Supplementary Tables S1-S9:

Table S1. Comparison of an exploratory PCA including all recorded behavioural variables and the PCA used in the main analyses. Comparison between an exploratory PCA including all recorded behavioural duration variables and the PCA used in the main analyses, which was based on biologically relevant vigilance-related duration measures.

	**Exploratory PCA (all behavioural variables)**	**PCA used in the main analyses**
Duration variables included	Not feeding, Ears forward, Ears Down, Ears asymmetric, Ears axial, Ears back up, Appraoching, Displacement, Scanning, Total response time	Not Feeding, Ears forward, Scanning, Total response time
Number of variables	10	4
Overall KMO	0.529	0.759
Bartlett's test of sphericity	χ^2 = 1891.578, df = 45, p < 0.001	χ^2 = 998.699, df = 6, p < 0.001
Eigenvalues	PC1 = 5.514; PC3 = 1.283; PC2 = 1.044	PC1 = 3.814
Variance explained among retained components (%)	PC1 = 43.2%; PC3 = 21.0%; PC2 = 14.2%	PC1 = 95.4%
Cumulative variance explained	78.40%	95.40%
Proportion explained among retained components	PC1 = 55.1%; PC3 = 26.8%; PC2 = 18.1%	PC1 = 100%
Highest component loadings on retained component(s)	PC1: Scanning = 0.928; Total response time = 0.905; Ears forward = 0.846	PC1: Ears forward = 0.996; Total response time = 0.987; Scanning = 0.981; Not feeding = 0.941
Highest loadings on PC3	Approaching = 0.858; Displacement = 0.735	NA
Highest loadings on PC2	Ears back up = 0.801; Ears axial = 0.718	NA
Communality range (h^2)	0.599–0.978	0.886–0.992
Uniqueness range (u^2)	0.022–0.401	0.008–0.114

Table S2. Distribution of playback stimulus categories across playback orders.

**Stimulus type**	**Order 1**	**Order 2**	**Order 3**	**Total**
Dove	8	10	17	35
Lion	12	11	12	35
Oxpecker	15	14	6	35

Table S3. Candidate model comparison for the overall playback response model examining giraffe vigilance responses across all playback categories. Candidate models were compared using Akaike's Information Criterion corrected for small sample size (AICc). K = number of estimated parameters; ΔAICc = difference in AICc relative to the best-supported model; AICcWt = Akaike weight; Cum.Wt = cumulative Akaike weight; LL = log-likelihood. Lower AICc values indicate stronger model support. Max VIF = maximum variance inflation factor among fixed effects within each model. Elevated VIF values in interaction models reflect expected correlation structures associated with higher-order interaction terms.

Model	Candidate model structure	Biological motivation	K	AICc	ΔAICc	AICcWt	Cum.Wt	LL	Max VIF
**m1**	PC ~ Stimulus Type + Order + Sex + Distance + (1 | Giraffe ID)	Main additive model including the core predictors of interest: stimulus type, playback order, sex, and playback distance.	9	250.15	0	0.34	0.34	−115.09	1.07
**m4**	PC ~ Stimulus Type * Sex + Order + Distance + (1 | Giraffe ID)	Tests whether the effect of stimulus type varies between sexes.	11	250.5	0.36	0.29	0.63	−112.78	10.71
**m2**	PC ~ Stimulus Type + Order + Sex + (1 | Giraffe ID)	Evaluates the core predictors without playback distance for comparison with the main additive model.	8	252.45	2.3	0.11	0.73	−117.45	1.06
**m5**	PC ~ Stimulus Type + Order + Sex + Distance + (1 | Giraffe ID) + (1 | Date)	Tests whether responses changed over the course of the study by including date-related variation as an additional random effect.	10	252.54	2.39	0.1	0.84	−115.06	1.07
**m6**	PC ~ Stimulus Type + Order + Sex + Distance + (1 | Giraffe ID) + (1 | Stimulus ID)	Tests whether responses vary according to the identity of the playback file used.	10	252.56	2.41	0.1	0.94	−115.07	1.07
**m3**	PC ~ Stimulus Type * Order + Sex + Distance + (1 | Giraffe ID)	Tests whether the effect of stimulus type varies depending on playback order.	13	254.91	4.76	0.03	0.97	−112.38	49.61
**m7**	PC ~ Stimulus Type + Order + Sex + Distance + (1 | Giraffe ID) + (1 | Date) + (1 | Stimulus ID)	Conservative control model including both date-related variation and playback-file identity as additional random effects.	11	255	4.86	0.03	1	−115.03	1.07
**m0**	PC ~ 1 + (1 | Giraffe ID)	Null reference model used to assess whether candidate predictors improve model fit beyond individual-level random variation.	3	294.7	44.56	<0.001	1	−144.23	–

Table S4. Playback distances (m) across stimulus categories and sexes in the overall playback response dataset. Values are presented as mean distance ± standard deviation (SD), together with observed distance ranges and sample sizes (N) for each stimulus type within females and males.

Sex	Stimulus type	Mean distance (m)	SD	Range (m)	N
Female	Dove	82.9	22.8	56–124	14
	Lion	80.3	25.5	47–125	16
	Oxpecker	75.4	28	39–124	16
Male	Dove	71.1	26.3	33–123	19
	Lion	70.2	21.7	44–123	19
	Oxpecker	70.1	26.1	33–133	19

Table S5. Candidate model comparison for the lion-oxpecker playback subset model examining giraffe vigilance responses. Candidate models tested the effects of stimulus type, playback order, sex, playback distance, and biologically motivated interaction terms, while accounting for repeated measures of individual giraffes using random intercepts for Giraffe ID. Models are ranked according to AICc support. K = number of estimated parameters; ΔAICc = difference in AICc relative to the best-supported model; AICcWt = Akaike weight; Cum.Wt = cumulative Akaike weight; LL = log-likelihood. Lower AICc values indicate stronger model support. Max VIF = maximum variance inflation factor among fixed effects within each model and was used to assess multicollinearity among predictors.

Model	Candidate model structure	Biological motivation	K	AICc	ΔAICc	AICcWt	Cum.Wt	LL	Max VIF
m1	PC ~ Stimulus Type + Order + Sex + Distance + (1 | Giraffe ID)	Main additive model including stimulus type, playback order, sex, and playback distance.	7	198.43	0	0.73	0.73	−91.31	1.04
m2	PC ~ Stimulus Type * Sex + Order + Distance + (1 | Giraffe ID)	Tests whether responses to lion and oxpecker stimuli differ between sexes.	8	200.46	2.03	0.27	1	−91.05	3.35
m0	PC ~ 1 + (1 | Giraffe ID)	Null reference model assessing whether fixed predictors improve model fit beyond individual-level random variation.	3	212.44	14.01	<0.001	1	−103.04	—

Table S6. Candidate model comparison for the carry-over response analysis examining vigilance responses to the second playback stimulus within lion-oxpecker playback sequences. Candidate models were compared using Akaike's Information Criterion corrected for small sample size (AICc). K = number of estimated parameters; ΔAICc = difference in AICc relative to the best-supported model; AICcWt = Akaike weight; Cum.Wt = cumulative Akaike weight; LL = log-likelihood. Lower AICc values indicate stronger model support.

Model	Candidate model structure	Biological motivation	K	AICc	ΔAICc	AICcWt	Cum.Wt	LL	Max VIF
**m1**	PC 2nd Stimulus ~ ID 2nd Stimulus + PC 1st Stimulus + Sex	Main additive carry-over model evaluating whether vigilance responses to the second playback stimulus varied according to the identity of the second stimulus, preceding response intensity, and sex.	5	107.21	0	0.52	0.52	−47.57	1.32
**m2**	PC 2nd Stimulus ~ ID 2nd Stimulus + PC 1st Stimulus + Sex + Interval	Evaluates whether inter-stimulus interval duration additionally influences vigilance responses to the second playback stimulus.	6	107.98	0.77	0.36	0.88	−46.49	1.48
**m3**	PC 2nd Stimulus ~ ID 2nd Stimulus * PC 1st Stimulus + Sex + Interval	Tests whether relationships between preceding and subsequent vigilance responses differ depending on second-stimulus identity.	7	111.03	3.82	0.08	0.95	−46.44	3.63
**m0**	PC 2nd Stimulus ~ 1	Null reference model used to assess whether carry-over predictors improve model fit beyond overall mean response variation.	2	112.09	4.88	0.05	1	−53.86	—

Table S7. Fixed effects of the carry-over response model additionally including inter-stimulus interval duration (model 2, see Table S5). The model evaluated whether vigilance responses to the second playback stimulus were associated with second-stimulus identity, preceding response intensity, sex, and the duration of the interval between playback stimuli. Fixed-effect estimates are shown relative to the reference categories second lion stimulus and female giraffes. Confidence intervals are presented as 95% CIs.

Fixed effects	Estimate	SE	95% CI	t	p
Intercept (2nd lion stimulus, Female)	1.869	0.494	0.860 to 2.879	3.782	<0.001
ID 2nd oxpecker relative to ID 2nd lion stimulus	-0.756	0.399	-1.572 to 0.060	-1.893	0.068
Preceding response (PC 1st stimulus)	0.348	0.221	-0.103 to 0.799	1.575	0.126
Male relative to Female	-0.978	0.364	-1.722 to -0.234	-2.684	0.012
Inter-stimulus interval duration	-0.004	0.003	-0.010 to 0.002	-1.383	0.177

Table S8. Fixed effects of the carry-over response model including an interaction between second-stimulus identity and preceding response intensity (model 3, see Table S5). The model evaluated whether the relationship between preceding and subsequent vigilance responses differed according to the identity of the second playback stimulus. Fixed-effect estimates are shown relative to the reference categories second lion stimulus and female giraffes. Confidence intervals are presented as 95% CIs.

Fixed effects	Estimate	SE	95% CI	t	p
Intercept (2nd lion stimulus, Female)	1.885	0.505	0.852 to 2.918	3.733	<0.001
2nd oxpecker relative to 2nd lion stimulus	-0.759	0.406	-1.589 to 0.071	-1.869	0.072
First-stimulus response intensity (PC 1st stimulus)	0.426	0.351	-0.292 to 1.144	1.213	0.235
Male relative to Female	-0.962	0.374	-1.728 to -0.196	-2.569	0.016
Inter-stimulus interval duration	-0.004	0.003	-0.010 to 0.002	-1.377	0.179
ID 2nd oxpecker × PC 1st stimulus	-0.12	0.414	-0.966 to 0.727	-0.289	0.775

Table S9. Results of Cox proportional hazards survival analyses examining latency to interrupt feeding ('Not Feeding') in response to playback stimuli. The shared frailty model included giraffe identity as a random frailty term to account for repeated measures, whereas the comparison model fixed frailty variance to zero. Estimates, standard errors (SE), t-values, and p-values are shown for fixed effects of stimulus type (oxpecker relative to lion), playback order (second relative to first playback stimulus), and sex (males relative to females). Frailty variance estimates and model fit statistics (restricted likelihood deviance and rAIC) are additionally provided for comparison between models.

**Predictor**	**Frailty model Estimate ± SE**	***t***	***p***	**No-frailty model Estimate ± SE**	***t***	***p***
Stimulus type (Oxpecker vs. Lion)	−0.297 ± 0.266	−1.117	0.264	−0.280 ± 0.257	−1.089	0.276
Playback order (Second vs. First)	−0.103 ± 0.263	−0.393	0.694	−0.095 ± 0.255	−0.373	0.709
Sex (Male vs. Female)	−0.197 ± 0.283	−0.697	0.486	−0.142 ± 0.248	−0.574	0.566
Giraffe-level frailty variance	0.132 ± 0.205	—	—	0 (fixed)	—	—
Restricted likelihood deviance	456.1	—	—	456.68	—	—
rAIC	458.1	—	—	456.68	—	—

Supplementary Figures S1-S3:

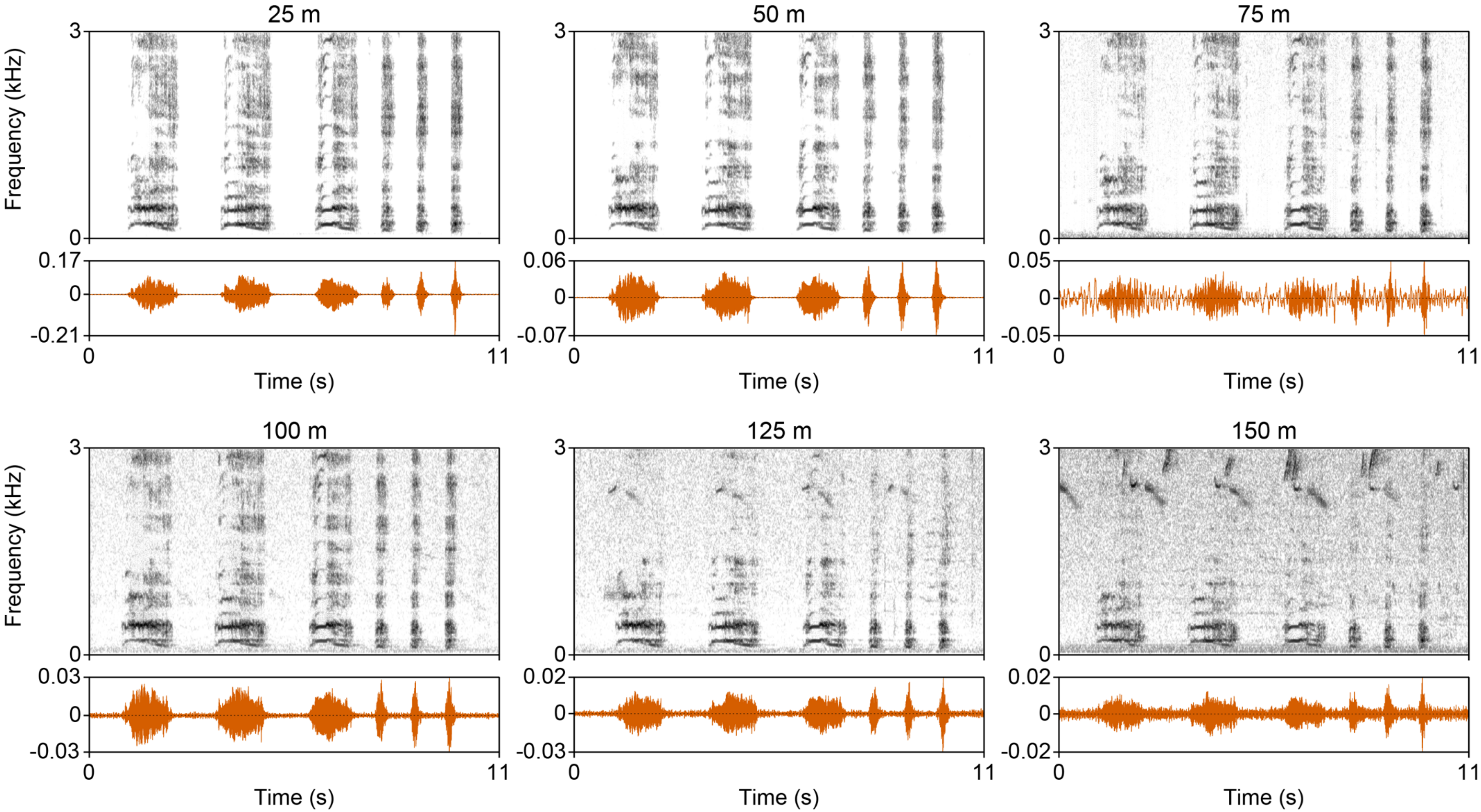

Figure S1. Spectrograms and waveforms of a lion roar-grunt sequence at different distances. Lion sequence consisting of three roars and three grunts, re-recorded at 25 m, 50 m, 75 m, 100, 125 m, and 150 m during propagation tests (FFT window = 0.07 s, dynamic range = 50 dB).

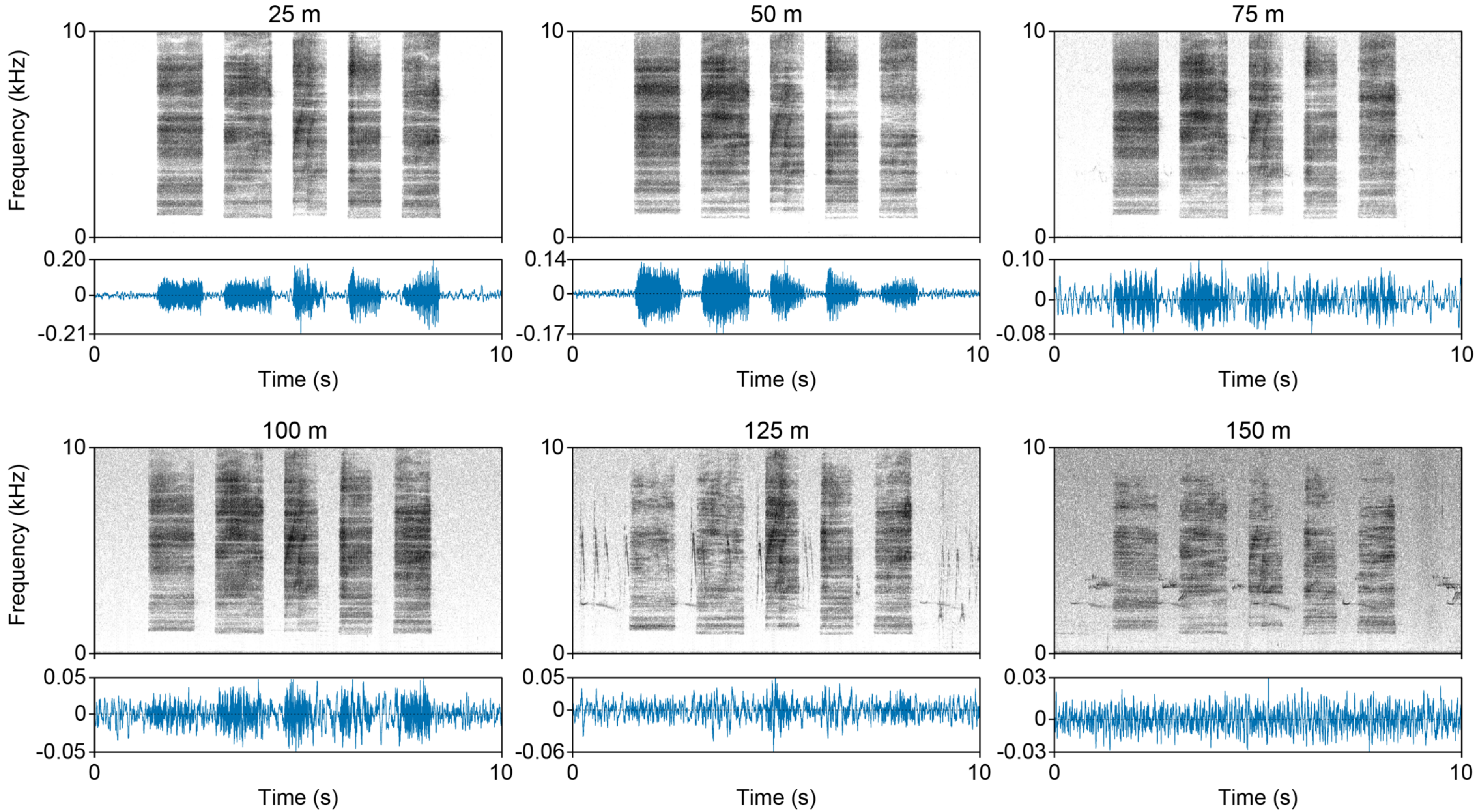

Figure S2. Spectrograms and waveforms of a red-billed oxpecker ‘ksss’ alarm call sequence at different distances. Red-billed oxpecker alarm sequence consisting of five ‘ksss’ calls, re-recorded at 25 m, 50 m, 75 m, 100, 125 m, and 150 m during propagation tests (FFT window = 0.04 s, dynamic range = 50 dB).

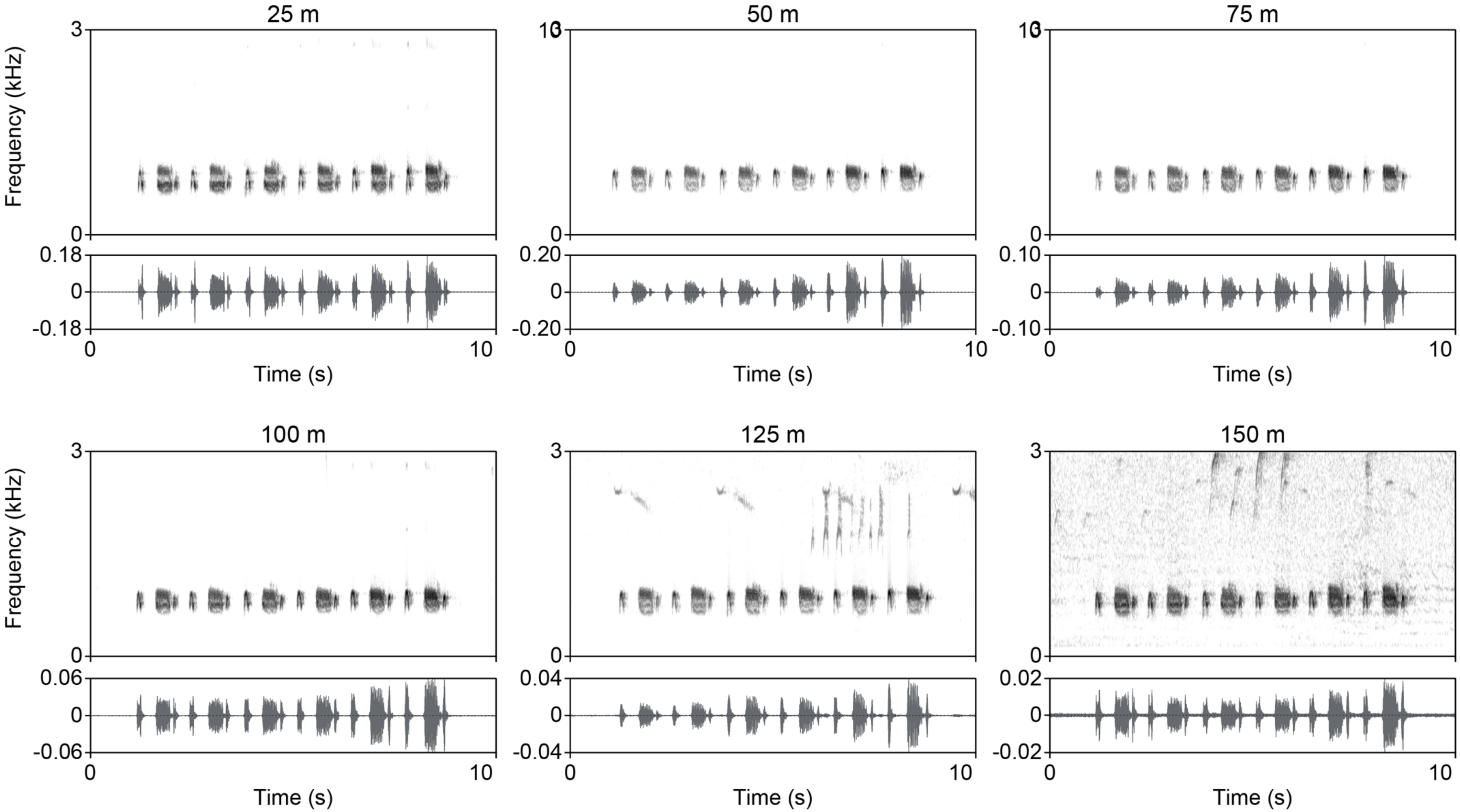

Figure S3. Spectrograms and waveforms of a ring-necked dove coo sequences at different distances. Rring-necked dove coo sequence consisting of six coo calls, re-recorded at 25 m, 50 m, 75 m, 100, 125 m, and 150 m during propagation tests (FFT window = 0.04 s, dynamic range = 50 dB).

References

Audacity Team. 2023. Audacity: Free audio editor and recorder [computer program]. Version 3.3, retrieved april 24, 2023 from https://www.Audacityteam.Org/.

Baker MC, Logue DM. 2007. A comparison of three noise reduction procedures applied to bird vocal signals. J Field Ornithol. 78(3):240-253. https://doi.org/10.1111/j.1557-9263.2007.00109.x.

Benedict L, Hardt B, Dargis L. 2021. Form and function predict acoustic transmission properties of the songs of male and female canyon wrens. Front Ecol Evol. 9(https://doi.org/10.3389/fevo.2021.722967.

Boersma P, Weenink D. 2023. Praat: Doing phonetics by computer [computer program]. Version 6.3.09, retrieved 10 march, 2023 from http://www.Praat.Org/.

Brenowitz EA. 1982. The active space of red-winged blackbird song. J Comp Physiol. 147(4):511-522. https://doi.org/10.1007/BF00612017.

den Hartog PM, Slabbekoorn H, ten Cate C. 2008. Male territorial vocalizations and responses are decoupled in an avian hybrid zone. Philos Trans R Soc Lond B Biol Sci. 363(1505):2879-2889. https://royalsocietypublishing.org/doi/abs/10.1098/rstb.2008.0046.

Elliott S. 1995. Xc590463. Accessible at www.Xeno-canto.Org/590463.

Lea AJ, Barrera JP, Tom LM, Blumstein DT. 2008. Heterospecific eavesdropping in a nonsocial species. Behav Ecol. 19(5):1041-1046. https://doi.org/10.1093/beheco/arn064.

Leite G. 2023a. Xc810692. Accessible at www.Xeno-canto.Org/810692.

Leite G. 2023b. Xc810974. Accessible at www.Xeno-canto.Org/810974.

McComb K, Packer C, Pusey A. 1994. Roaring and numerical assessment in contests between groups of female lions, *panthera leo*. Anim Behav. 47(2):379-387. https://www.sciencedirect.com/science/article/pii/S0003347284710529.

Moyer MJ et al. 2024. Sex-specific responses to simulated territorial intrusions provide evidence for relaxed selection pressure on female song in orchard orioles. J Field Ornithol. 7 95(3). https://journal.afonet.org/vol95/iss3/art7/.

Rudman L. 2022a. Xc718396. Accessible at www.Xeno-canto.Org/718396.

Rudman L. 2022b. Xc718397. Accessible at www.Xeno-canto.Org/718397.
